# Trends in rehabilitation needs for neurological disorders in China, 1990–2021: a cross-sectional analysis of the Global Burden of Disease Study 2021

**DOI:** 10.3389/fmed.2026.1688298

**Published:** 2026-04-09

**Authors:** Chengcheng Zhang, Yuqi Xiu, Wenjuan Ying, Yifeng Xiao

**Affiliations:** 1 The First Affiliated Hospital of Shantou University Medical College, Shantou, Guangdong, China; 2 School of Public Health, Harbin Medical University, Harbin, Heilongjiang, China; 3 Shantou University Medical College, Shantou, Guangdong, China

**Keywords:** epidemiology, Global Burden of Disease, neurological disorders, public health, rehabilitation needs

## Abstract

**Background:**

Neurological disorders are a leading cause of long-term disability, generating substantial rehabilitation needs. China’s rapid population aging and evolving epidemiological profile underscore the urgency of quantifying these needs.

**Method:**

Using data from the Global Burden of Disease Study 2021, we assessed rehabilitation needs for 10 neurological disorders in China from 1990 to 2021. Prevalence and years lived with disability (YLDs) were analyzed by age, sex, and cause, benchmarked against global trends. Temporal trends were quantified by estimated annual percentage change (EAPC), and Bayesian age–period–cohort modeling was applied to forecast to 2050.

**Results:**

From 1990 to 2021, China’s age-standardized prevalence and YLDs rates increased significantly, with EAPCs of 0.42 (95% CI 0.38 to 0.45) and 0.40 (95% CI 0.36 to 0.43), both exceeding global averages. The largest absolute burdens in 2021 were from stroke, Alzheimer’s disease, and Parkinson’s disease. Parkinson’s disease (EAPC = 1.85, 95% CI 1.78 to 1.92), multiple sclerosis (1.42, 95% CI 1.36 to 1.48), and motor neuron disease (1.11, 95% CI 1.05 to 1.17) showed the steepest proportional rises. Women bore higher late-life burdens, while men had greater trauma-related disability. Rehabilitation needs were concentrated in older adults, with substantial geographic and service-access inequities reported in prior national surveys. Forecasts to 2050 indicate sustained growth, with neurodegenerative disorders comprising an increasing share of total rehabilitation demand.

**Conclusion:**

The scale and pace of growth in China’s neurological rehabilitation needs reflect demographic aging, improved survival, and persistent service gaps. Meeting this challenge will require decentralizing rehabilitation, integrating disease-specific pathways into universal health coverage, and prioritizing underserved rural and older populations.

## Introduction

Neurological disorders constitute one of the foremost global challenges in public health, ranking among the leading causes of disability and dependency across the life course (1). Unlike many acute conditions, neurological diseases such as stroke, Alzheimer’s disease, Parkinson’s disease, traumatic brain injury, and multiple sclerosis often result in enduring impairments of motor function, cognition, speech, and self-care (2). These long-term consequences translate into substantial rehabilitation needs, both at the individual and systems levels, that persist well beyond the acute phase of treatment (1, 3). Despite their growing burden, the rehabilitation implications of neurological disorders have historically been underrepresented in epidemiological surveillance, health system planning, and global health financing priorities (4).

Recent years have seen increasing recognition of rehabilitation as an essential component of universal health coverage, endorsed by WHO’s Rehabilitation 2030 initiative (4). Yet, a critical gap remains in quantifying the scale and evolution of rehabilitation needs specifically attributable to neurological disorders, particularly in low- and middle-income countries undergoing demographic transition (1, 5). In China, prior burden-of-disease studies using GBD estimates have provided important evidence that neurological disorders contribute substantially–and increasingly–to national disability burden across recent decades, including analyses covering 1990–2019 and updated assessments based on GBD 2021 data with national and provincial estimates (6, 7). However, these studies have largely focused on overall disease burden metrics (e.g., prevalence, DALYs, mortality) and have not explicitly translated neurological disability into rehabilitation needs or projected the future trajectory of rehabilitation demand.

Among them, China faces a uniquely complex scenario: with a rapidly aging population and the world’s largest number of adults aged ≥ 60 years, the country is witnessing a surge in age-related neurological morbidity (8). Although acute-phase care (e.g., thrombolysis, neurosurgery) has expanded in recent decades, the capacity of China’s rehabilitation system–already marked by urban–rural disparities, personnel shortages, and limited community-based services–lags far behind growing demand (9). As survival improves and the population living with long-term neurological impairment accumulates, the mismatch between rehabilitation demand and service capacity is likely to widen, underscoring the need for nationally comparable, time-trend evidence to support strategic planning, workforce development, and resource allocation. National estimates of neurological rehabilitation needs are urgently needed to inform policy prioritization, resource allocation, and workforce development.

In response to these unmet needs, this study aims to comprehensively assess the rehabilitation burden associated with neurological disorders in China using data from the Global Burden of Disease (GBD) Study 2021 (10). By analyzing long-term trends from 1990 to 2021 in prevalence and years lived with disability (YLDs), disaggregated by age, sex, and condition, and benchmarking them against global patterns, we seek to generate robust, policy-relevant evidence to guide rehabilitation planning in the next phase of health system development.

## Materials and methods

### Study design and data sources

This study presents a comprehensive cross-sectional and longitudinal analysis of rehabilitation needs due to neurological disorders in China, drawing on data from the GBD 2021, coordinated by the Institute for Health Metrics and Evaluation. The GBD framework integrates over 100,000 data sources globally, including vital registration systems, population-based surveys, disease registries, hospital discharge records, and peer-reviewed studies, to generate standardized and comparable estimates of disease burden across countries and time (11).

For this analysis, we specifically utilized data from the GBD Rehabilitation database—a curated subset of the GBD platform that systematically quantifies the burden of conditions amenable to rehabilitation interventions. This database provides internally consistent estimates for 25 priority conditions identified by WHO as rehabilitation-relevant. Of these, 10 neurological disorders were included in our study based on their potential to cause long-term functional impairment and documented responsiveness to rehabilitation: Alzheimer’s disease and other dementias, cerebrovascular disease (stroke), epilepsy, Parkinson’s disease, multiple sclerosis, motor neuron disease, cerebral palsy, traumatic brain injury, spinal cord injury, and other neurological disorders. These conditions were defined according to standardized ICD-10 code groupings and mapped to GBD cause categories (3, 11).

Our analysis focuses on trends in prevalence and years lived with disability (YLDs) from 1990 to 2021, disaggregated by age, sex, and condition. Estimates for China were extracted directly from the publicly accessible GBD Compare and Global Health Data Exchange (GHDx) platforms. The study adheres to GBD’s established protocols for data quality assurance, Bayesian meta-regression modeling, and uncertainty quantification, ensuring robust and replicable estimates of rehabilitation need over time (12).

### Bayesian age-period-cohort model

To evaluate temporal trends and forecast future burden of neurological disorders, we applied a Bayesian age–period–cohort (BAPC) model. This modeling approach disaggregates disease patterns into three principal components:

Age effect (α): Captures variations in disease burden attributable to biological aging and age-related exposure.

Period effect (β): Reflects influences that occur at a specific time period affecting all age groups, such as advancements in medical technology, diagnostic access, and public health interventions.

Cohort effect (γ): Represents generational risk differences, accounting for shifts in exposure profiles or early-life determinants across birth cohorts.

The BAPC model is mathematically expressed as:


$$Y_{i\,j\,k}=\mu +\alpha_{i}+\beta_{j}+\gamma_{k}+\varepsilon_{i\,j\,k}$$

where Y_*ijk*_ represents the observed disease burden for age group i, period j, and cohort k; is the overall intercept; and ϵ_*ijk*_ is the random error term.

Model fitting was performed via Markov Chain Monte Carlo sampling, generating posterior distributions for each component. Convergence diagnostics, including the Gelman–Rubin statistic, were used to ensure stability and reliability of the estimates. This framework provides a probabilistic basis for projecting age-standardized prevalence and disability rates to 2050, integrating both historical patterns and demographic dynamics (13, 14).

### Statistical analysis

To quantify the burden of rehabilitation needs attributable to neurological disorders, we employed a series of age-standardized indicators and temporal trend analyses based on data from the GBD 2021. The age-standardized rate (ASR) was calculated using the direct standardization method to adjust for differences in age structure across populations (15). The formula used was:


$$A\,S\,R=\frac{\sum_{i=1}^{N}a_{i}\,w_{i}}{\sum_{i=1}^{N}w_{i}}$$

Where *a_i_* is the age-specific rate in the *i^th^* age group, *w_i_* is the population weight for the same age group in the standard population, and *N* is the total number of age groups.

Two primary indicators were derived using ASR to capture rehabilitation needs. The ASPR per 100,000 population represented the number of individuals living with a neurological disorder requiring rehabilitation, providing a measure of the overall magnitude of rehabilitation demand. The age-standardized YLDs rate per 100,000 population quantified nonfatal health loss associated with these disorders, weighted by disability severity and comorbidity, thereby reflecting the intensity of rehabilitation needs over time.

To assess long-term trends from 1990 to 2021, we calculated the EAPC in ASPR and YLDs rates using a log-linear regression model:


l⁢n⁢(A⁢S⁢R)=α+β×y⁢e⁢a⁢r+ε

where β represents the annual rate of change. The EAPC and its 95% confidence interval (CI) were then calculated as:


E⁢A⁢P⁢C=100×[e⁢x⁢p⁢(β)-1]

A statistically significant increasing trend was defined as an EAPC > 0 with a 95% CI entirely above zero; a decreasing trend as an EAPC < 0 with a 95% CI entirely below zero; and a stable trend when the 95% CI included zero. This method allowed us to robustly assess the temporal dynamics of rehabilitation needs for neurological disorders burden across populations (16).

All estimates are presented with 95% uncertainty intervals (UI) derived from 1,000 posterior draws. Analyses were conducted using R version 4.2.2.

## Results

### Overall trends in rehabilitation needs for neurological disorders: China vs. global, 1990–2021

**FIGURE 1:**
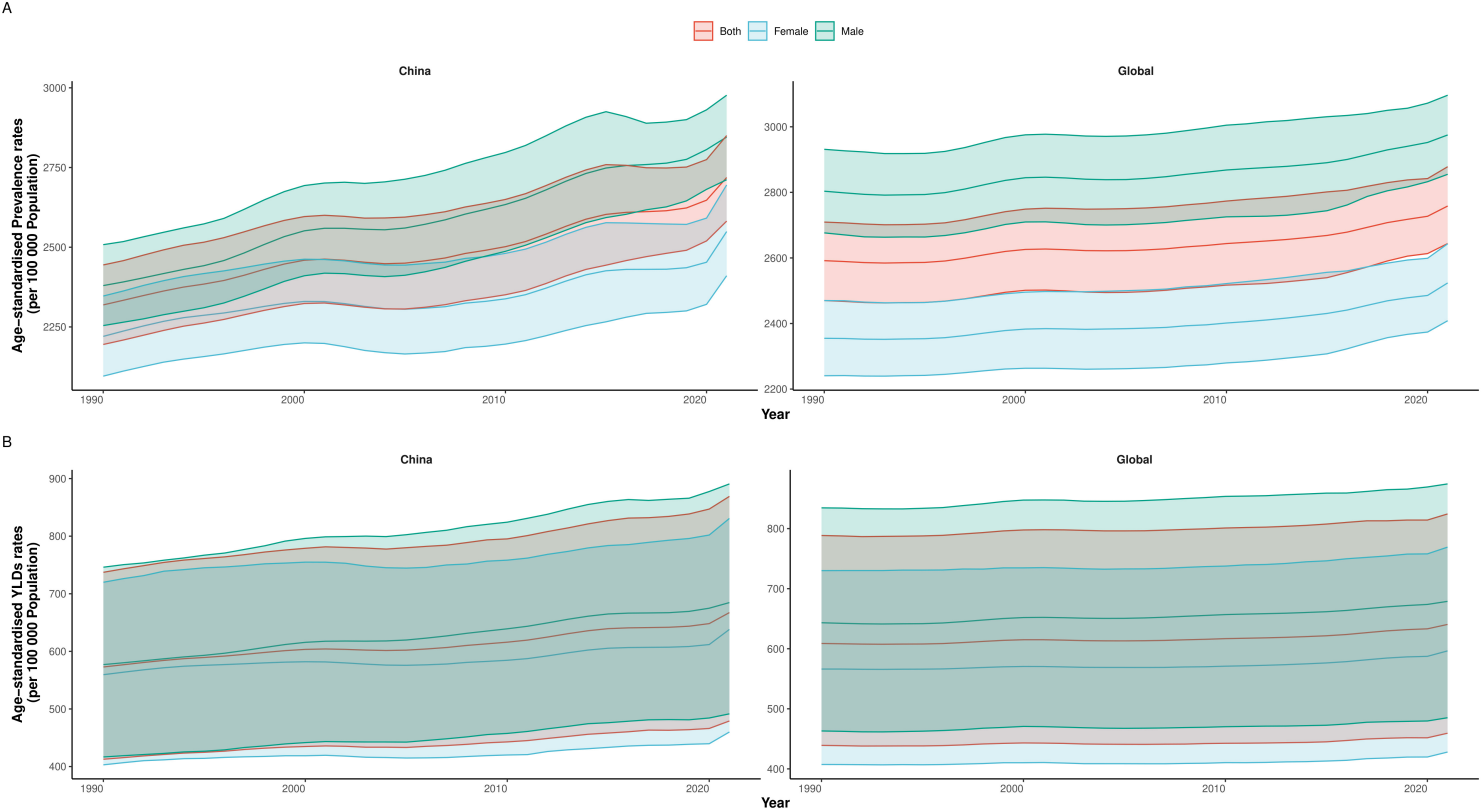
Trends in age-standardized rehabilitation needs for neurological disorders by sex in China and globally, 1990–2021. **(A)** Age-standardized prevalence rate. **(B)** Age-standardized YLDs rate. YLDs, years lived with disability.

From 1990 to 2021, rehabilitation needs due to neurological disorders increased markedly both in China and globally, with China demonstrating a sharper absolute and relative rise. In China, the number of individuals requiring rehabilitation surged from 20.85 million (95% UI 19.75–22.05) in 1990 to 50.55 million (47.77–53.15) in 2021, a relative increase of 142% (Table 1). In contrast, the global total rose from 114.14 million (108.68–119.70) to 225.38 million (215.84–235.21), a 97% increase over the same period. China’s ASPR increased from 2319.09 (2,194.96–2,444.43) to 2,718.60 (2,581.84–2,850.28) per 100,000 population, with an EAPC of 0.42 (0.38–0.45). Sex-specific patterns were comparable: ASPR among males rose from 2,379.69 to 2,845.64, and among females from 2,220.34 to 2,549.12 per 100,000 (Figure 1). By comparison, the global ASPR showed slower growth, rising from 2,591.71 (2,469.95–2,709.35) to 2,758.37 (2,644.02–2,878.23) per 100,000 (EAPC 0.17 [0.15–0.19]) (Table 1), with male and female rates increasing from 2,803.53 to 2,975.10 and 2,354.86 to 2,543.73, respectively (Figure 1).

**TABLE 1:** The number of prevalence cases and the ASPR of rehabilitation needs for neurological disorders in 1990 and 2021, and its temporal trends from 1990 to 2021 in China and global.

Location	Disease	1990		2021		1990–2021
		Number of cases (95% UI)	ASPR/100,000 (95% UI)	Number of cases (95% UI)	ASPR/100,000 (95% UI)	EAPC (95% CI)
Global	Neurological disorders	114,139,977 (108,682,395, 119,703,375)	2,591.71 (2,469.95, 2,709.35)	225,382,920 (215,836,356, 235,207,819)	2,758.37 (2,644.02, 2,878.23)	0.13 (0.11, 0.15)
	Cerebral palsy	29,963,184 (25,882,228, 34,466,037)	529.22 (457.56, 608.42)	63,458,010 (58,211,346, 69,497,425)	827.46 (758.81, 906.56)	1.61 (1.56, 1.67)
	Cerebrovascular disease (stroke)	26,235,171 (24,836,412, 27,753,474)	640.81 (605.94, 679.73)	50,633,759 (47,857,832, 53,732,751)	595.39 (563.49, 631.06)	−0.31 (−0.33, −0.29)
	Traumatic brain injury	24,699,677 (23,817,961, 25,796,974)	535.63 (516.42, 559.29)	37,861,891 (36,272,057, 39,700,733)	447.26 (428.61, 468.91)	−0.66 (−0.70, −0.62)
	Alzheimer’s disease and dementia	22,608,953 (19,819,623, 25,453,903)	686.61 (607.00, 771.74)	58,344,135 (51,211,541, 65,857,434)	709.02 (622.76, 801.13)	−0.01 (−0.04, 0.01)
	Spinal cord injury	10,811,245 (9,929,689, 11,831,208)	222.51 (205.41, 241.40)	15,388,832 (13,999,313, 17,060,722)	183.42 (166.84, 203.53)	−1.00 (−1.04, −0.95)
	Parkinson’s disease	1,454,319 (1,231,851, 1,702,161)	39.85 (33.76, 46.51)	5,433,273 (4,668,994, 6,354,622)	64.01 (55.03, 74.90)	1.52 (1.49, 1.54)
	Multiple sclerosis	773,103 (671,480, 896,799)	17.13 (14.91, 19.73)	1,452,654 (1,294,704, 1,643,042)	17.06 (15.19, 19.30)	0.08 (0.04, 0.11)
	Motor-neuron disease	139,050 (117,694, 162,595)	2.88 (2.46, 3.36)	234,201 (202,558, 269,298)	2.84 (2.46, 3.26)	0.11 (0.03, 0.18)
	Guillain–Barré syndrome	96,819 (77,059, 121,120)	1.93 (1.54, 2.38)	471,842 (389,181, 554,135)	5.91 (4.87, 6.97)	0.95 (0.26, 1.64)
	Neural tube defects	833,904 (683,788, 997,329)	14.13 (11.58, 16.87)	927,739 (784,927, 1,085,546)	12.58 (10.66, 14.71)	−0.35 (−0.40, −0.31)
China	Neurological disorders	20,845,976 (19,748,134, 22,050,999)	2,319.09 (2,194.96, 2,444.43)	50,549,986 (47,771,257, 53,148,819)	2,718.60 (2,581.84, 2,850.28)	0.42 (0.38, 0.45)
	Cerebral palsy	3,817,602 (3,129,108, 4,809,178)	310.20 (253.96, 391.04)	5,383,657 (4,976,113, 5,801,851)	405.53 (375.03, 437.31)	1.21 (1.09, 1.34)
	Cerebrovascular disease (stroke)	6,329,695 (5,877,428, 6,841,602)	710.38 (653.30, 772.49)	16,323,989 (14,949,186, 17,744,135)	807.65 (742.93, 874.63)	0.41 (0.38, 0.45)
	Traumatic brain injury	4,973,632 (4,774,001, 5,222,002)	473.11 (454.02, 496.81)	9,269,581 (8,829,683, 9,774,994)	481.00 (459.59, 505.01)	−0.11 (−0.24, 0.02)
	Alzheimer’s disease and dementia	4,226,680 (3,645,425, 4,812,387)	716.64 (625.02, 815.69)	17,712,347 (15,307,296, 20,066,685)	924.31 (801.69, 1,045.23)	0.48 (0.41, 0.56)
	Spinal cord injury	1,693,396 (1,573,323, 1,842,856)	149.76 (139.65, 162.03)	2,764,365 (2,556,346, 3,005,363)	151.59 (140.31, 164.87)	−0.34 (−0.60, −0.07)
	Parkinson’s disease	301,118 (246,531, 365,138)	42.39 (34.46, 51.21)	2,342,680 (1,943,148, 2,835,877)	113.37 (94.04, 137.55)	3.16 (3.03, 3.29)
	Multiple sclerosis	13,765 (10,265, 18,210)	1.20 (0.92, 1.56)	30,905 (24,687, 38,501)	1.71 (1.34, 2.18)	0.95 (0.79, 1.11)
	Motor-neuron disease	22,062 (17,428, 27,447)	1.83 (1.48, 2.24)	28,632 (23,218, 34,710)	1.97 (1.58, 2.40)	0.28 (0.22, 0.35)
	Guillain–Barré syndrome	6,398 (4,668, 8,660)	0.58 (0.43, 0.75)	9,387 (6,967, 12,260)	0.62 (0.47, 0.80)	0.39 (0.20, 0.57)
	Neural tube defects	87,607 (67,240, 112,161)	7.24 (5.56, 9.26)	58,826 (48,800, 70,682)	5.45 (4.53, 6.54)	0.10 (−0.57, 0.79)

ASPR, age-standardized prevalence rate; EAPC, estimate annual percentage change.

YLDs trends mirrored those of ASPR (Supplementary Table 1 and Figure 1). China’s total YLDs nearly tripled, from 5.11 million (3.67–6.62) to 12.41 million (8.91–16.15), while the age-standardized YLDs rate rose from 572.88 (412.68–737.27) to 667.36 (479.14–869.11) per 100,000 (EAPC 0.40 [0.36–0.43]). Sex-stratified YLDs rates exhibited consistent increases. Globally, total YLDs grew from 26.65 million (19.18–34.60) to 52.35 million (37.57–67.46), and the rate increased from 608.80 (439.13–788.52) to 640.50 (459.39–824.44) per 100,000 (EAPC 0.13 [0.11–0.15]). The faster growth in China underscores a widening gap in rehabilitation needs over time.

### Cause-specific burden trends of rehabilitation needs for neurological disorders: China vs. global, 1990–2021

**FIGURE 2:**
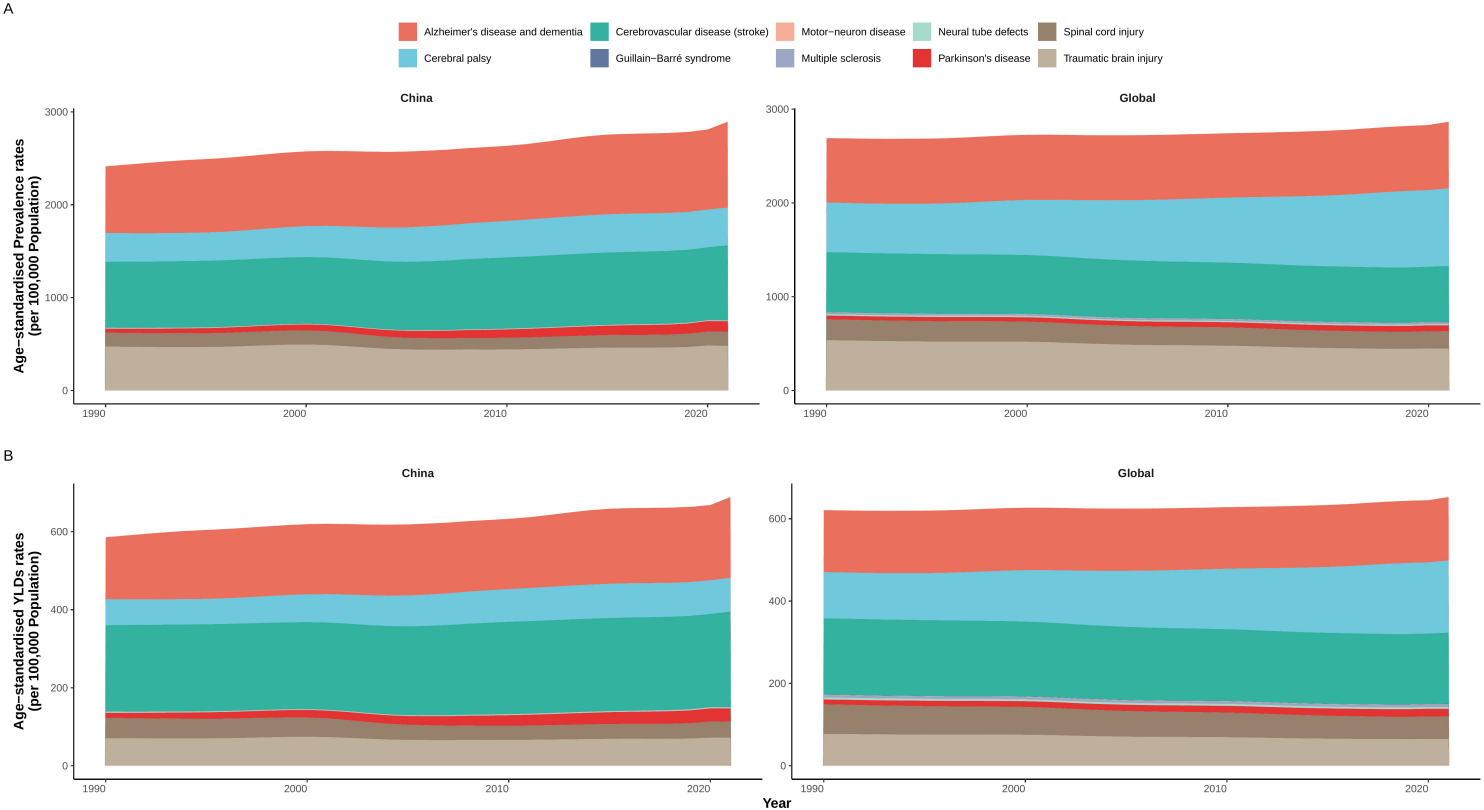
Cause-specific trends in age-standardized rehabilitation needs for neurological disorders in China and globally, 1990–2021. **(A)** Age-standardized prevalence rate. **(B)** Age-standardized YLDs rate. YLDs, years lived with disability.

Between 1990 and 2021, the ASPR of most neurological disorder subtypes increased in China, with Parkinson’s disease exhibiting the steepest rise—from 42.39 (95% UI 34.46–51.21) to 113.37 (94.04–137.55) per 100,000 (Figure 2 and Table 1). In contrast, the most pronounced global increase was observed for Guillain–Barré syndrome, which rose from 1.93 (1.54–2.38) to 5.91 (4.87–6.97) per 100,000. Alzheimer’s disease and other dementias increased notably faster in China (from 716.64 [625.02–815.69] to 924.31 [801.69–1,045.23]) than globally (from 686.61 to 709.02 per 100,000). Cerebral palsy showed an upward trend in both settings, with a sharper rise globally (from 529.22 [457.56–608.42] to 827.46 [758.81–906.56]) compared to China (from 310.20 [253.96–391.04] to 405.53 [375.03–437.31]). Stroke prevalence rose in China from 710.38 (653.30–772.49) to 807.65 (742.93–874.63), while it declined globally (640.81 to 595.39 per 100,000). Traumatic brain injury and spinal cord injury increased modestly in China (481.00 and 151.59 per 100,000 in 2021) but declined globally (447.26 and 183.42). Multiple sclerosis and motor neuron disease showed mild growth in China (to 1.71 and 1.97 per 100,000) but remained stable worldwide. Only neural tube defects declined in both settings, with a more pronounced decrease in China (from 7.24 to 5.45 per 100,000).

Trends in age-standardized YLDs rate largely paralleled prevalence patterns (Figure 2 and Table 1). In China, Parkinson’s disease YLDs increased from 12.66 (8.54–17.40) to 33.78 (23.35–45.84) per 100,000, while Guillain–Barré syndrome had the largest global increase (from 0.57 [0.37–0.86] to 1.75 [1.12–2.53]). Alzheimer’s disease YLDs rose more substantially in China (from 158.70 [109.28–208.07] to 207.27 [144.04–271.71]) than globally (150.23 to 154.18). Cerebral palsy YLDs rose more steeply globally (from 112.34 [76.87, 155.22] to 175.14 [120.28–234.45]) than in China (from 66.36 [44.97, 97.17] to 86.45 [60.48–115.57]). Stroke YLDs increased in China but declined globally. YLDs due to spinal cord injury and traumatic brain injury declined globally, but remained stable or slightly increased in China. Neural tube defects showed a consistent decline in both regions, again more marked in China.

### Age- and sex-specific burden of rehabilitation needs for neurological disorders in China, 2021

**FIGURE 3:**
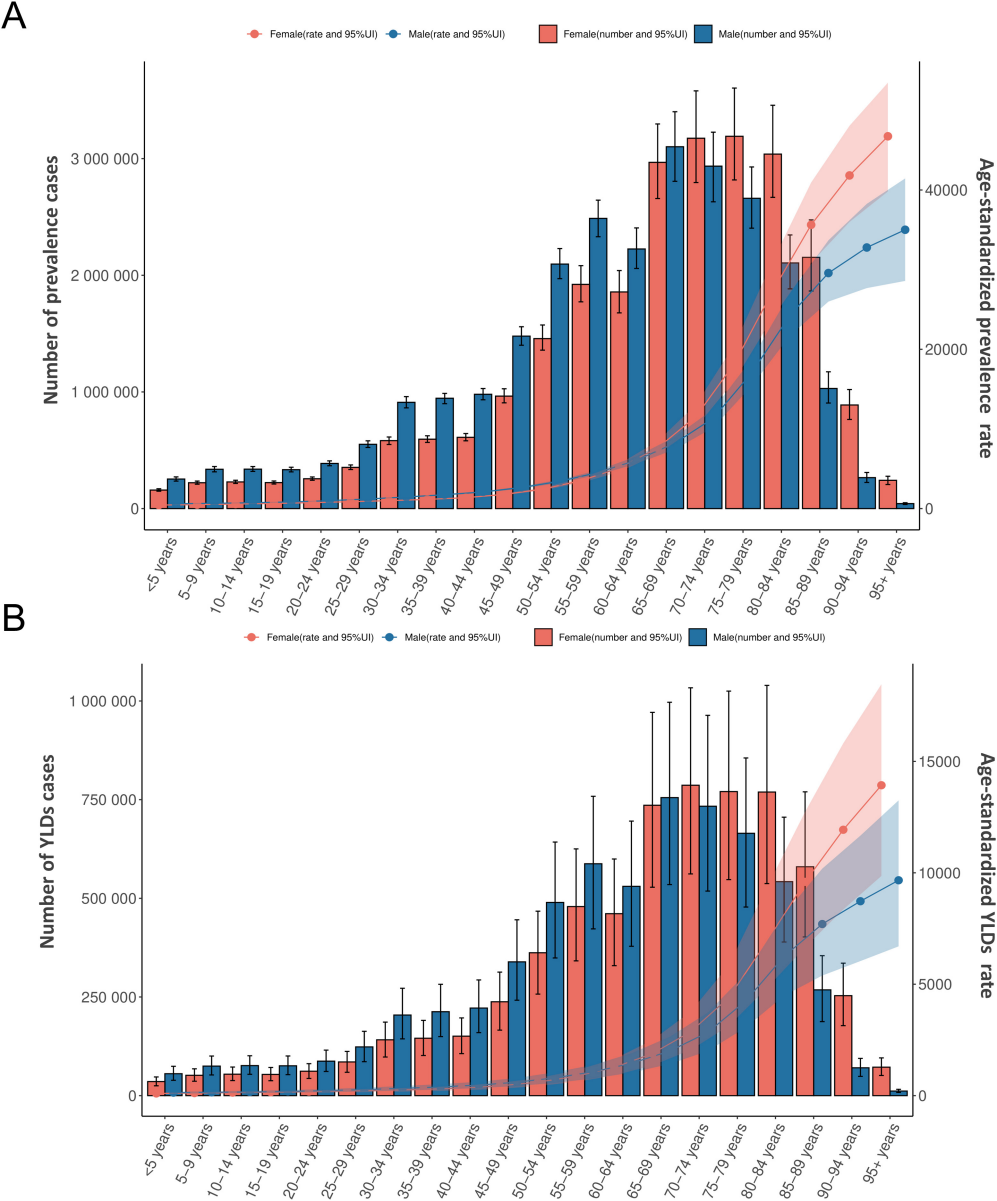
The age- and sex-specific burden of rehabilitation needs for neurological disorders in China in 2021: number of cases and age-standardized prevalence **(A)** and YLDs rates **(B)**. YLDs, years lived with disability.

In 2021, the burden of neurological disorders in China exhibited a distinct age gradient and significant sex-specific differences in both prevalence and YLDs (Figure 3). For prevalent cases, males showed an age-related increase, peaking at 3.10 million (95% UI: 2.81–3.40) in the 65–69-year group before declining in older age, whereas females reached a higher peak of 3.19 million (2.82–3.61) in the 75–79-year group, followed by a similar decline. A parallel pattern was observed for YLDs: males had the highest YLDs burden at 755,252 (534,796–996,764) in the 65–69-year group, while females peaked later at 786,564 (561,833–1,033,409) in the 70–74-year group. For both sexes, prevalence and YLDs counts rose steadily with age up to these peaks, reflecting a consistent late-life burden maximum.

Age-specific rates increased continuously with age and revealed notable sex disparities. In males, the ASPR surged from 608.05 (95% UI: 565.32–652.43) per 100,000 in children aged < 5 years to 35,009.49 (28,585.10–41,455.76) per 100,000 in those aged ≥ 95 years, with a corresponding YLDs rate increase from 133.58 (93.55–178.63) to 9,665.66 (6,700.09–13,250.74) per 100,000. Among females, the prevalence rate escalated more sharply across the same age spectrum, from 440.27 (412.55–471.45) to 46,746.66 (39,815.09–53,472.88) per 100,000, with age-standardized YLDs rate following suit (99.03 [69.21–131.48] to 13,930.84 [9,859.42–18,460.64] per 100,000). Critically, females exhibited slightly higher rates of both prevalence and disability than males across nearly all age groups, with the gap widening in the oldest age strata.

### Cause-specific burden of rehabilitation needs for neurological disorders in China, 2021

In 2021, the burden of neurological disorders in China displayed marked sex-specific and age-specific variation (Supplementary Tables 2, 3). Males exhibited a higher overall ASPR than females (2,845.64 [95% UI 2,711.73–2,976.81] vs. 2,549.12 [2,410.59–2,695.38] per 100,000), and a faster upward trend (EAPC 0.54 [0.52–0.57] vs. 0.30 [0.26–0.34]), although females accounted for slightly higher total numbers of prevalent cases and YLDs. Traumatic brain injury showed the most pronounced male predominance, with a two-fold higher ASPR (641.46 [611.49–674.04] vs. 316.75 [301.85–332.87]) and total cases (6.18 million vs. 3.09 million), alongside a stable trend in males (EAPC 0.04 [−0.07 to 0.16]) and a declining trend in females (−0.34 [−0.49 to −0.18]). Conversely, Alzheimer’s disease and other dementias disproportionately affected females, who had a 30% higher prevalence rate (1,053.44 [922.37–1,189.29] vs. 753.60 [642.39–863.24]) and nearly twice the number of cases (11.22 million vs. 6.49 million). Parkinson’s disease was more common in males (141.44 [116.71–172.08] vs. 90.32 [75.66–109.57]), with faster growth (EAPC 3.44 vs. 2.75). Stroke showed near-equal prevalence between sexes, but a steeper increase in males (EAPC 0.57 vs. 0.20). Spinal cord injury declined in both sexes, with a sharper decrease among females (EAPC −1.50 vs. −1.00). Guillain–Barré syndrome was slightly more prevalent in females and showed faster female growth, while neural tube defects displayed minimal sex differences.

**FIGURE 4:**
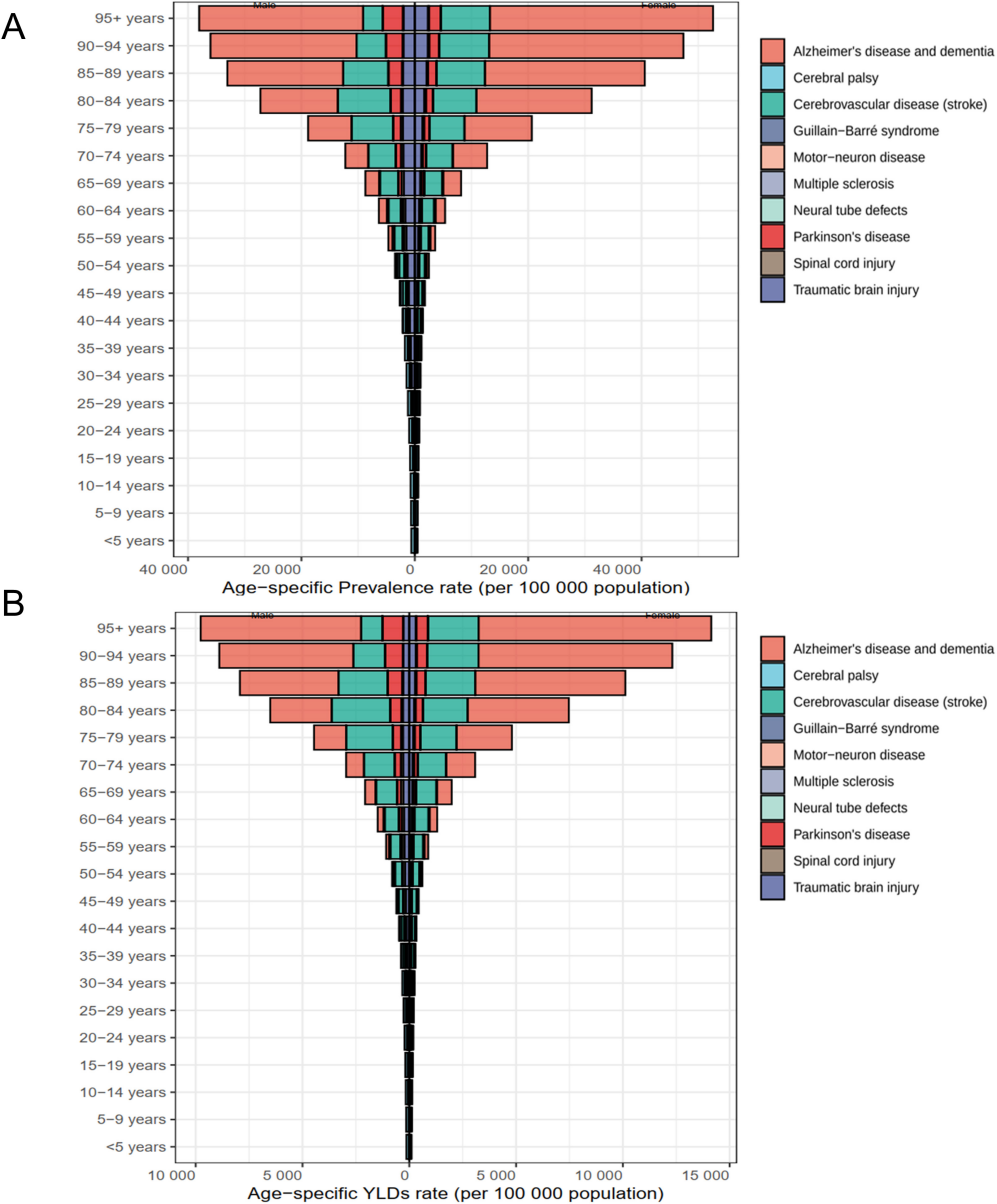
The distribution of age-standardized prevalence **(A)** and YLDs **(B)** rates across rehabilitation needs for neurological disorders by age and sex in China in 2021. YLDs, years lived with disability.

Age-specific patterns varied considerably (Figure 4). Among children aged 10–14 years, cerebral palsy was the leading contributor to both prevalence and disability, particularly among males (538.21 and 115.52 per 100,000 vs. 381.57 and 81.91 in females). In working-age adults (40–64 years), stroke was the dominant condition, with prevalence exceeding 2,100 per 100,000 and YLDs approaching 660 per 100,000 in the 60–64-year group. In adults aged ≥ 75 years, the burden shifted to Alzheimer’s disease and dementias, especially among females aged 85–89 years, who exhibited exceptionally high rates (28,164.20 per 100,000 prevalence; 7,028.75 YLDs). Parkinson’s disease also rose sharply with age, becoming a major contributor from age 65 onward, and peaking among the oldest adults.

### Projected burden of rehabilitation needs for neurological disorders in China by 2050

**FIGURE 5:**
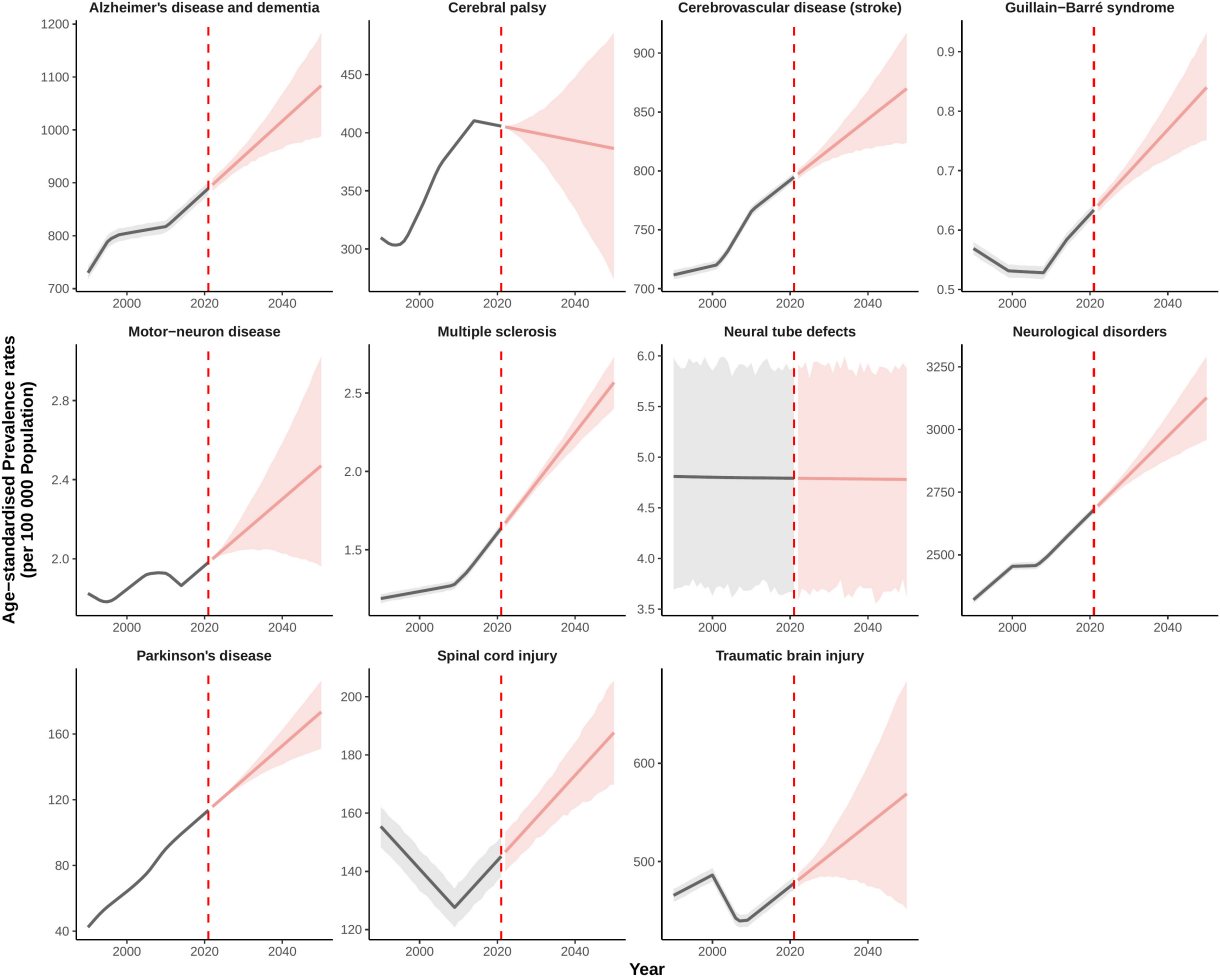
Projected trends in age-standardized prevalence rate of rehabilitation needs for neurological disorders in China, 1990–2050.

From 2021 to 2050, the ASPR of neurological disorders in China is projected to rise substantially, continuing the upward trajectory observed over the past three decades (Figure 5). The ASPR for all neurological disorders is expected to increase from 2,679.88 per 100,000 population (95% UI: 2,665.74–2,694.30) in 2021 to 3,126.64 (2,957.37–3,290.44) by 2050, representing a 16.67% relative increase. Among specific conditions, the most pronounced projected increase is observed for multiple sclerosis, with ASPR expected to rise from 1.64 (1.61–1.67) in 2021 to 2.57 (2.40–2.73) in 2050—a 56.70% increase. Similarly, Parkinson’s disease is projected to increase markedly from 113.69 (113.58–113.79) to 173.52 (151.30–192.49), corresponding to a 52.62% rise. Other conditions projected to undergo significant increases in ASPR include Guillain–Barré syndrome, rising from 0.63 (95% UI: 0.62–0.64) in 2021 to 0.84 (0.75–0.93) in 2050, reflecting a 32.53% increase; spinal cord injury, increasing from 145.18 (138.02–152.19) to 187.69 (169.85–205.51), a 29.28% rise; and motor-neuron disease, which is projected to grow from 1.98 (1.97–1.99) to 2.47 (1.96–3.02), representing a 24.65% increase. In contrast, while conditions such as stroke, Alzheimer’s disease and other dementias, and traumatic brain injury are expected to maintain high absolute prevalence, their relative growth in age-standardized rates is projected to be more modest over the same period.

## Discussion

Over the past three decades, the rehabilitation needs for neurological disorders in China have increased more sharply than in most other countries, surpassing both the global mean and the regional average for upper-middle-income economies (1, 17). This pattern reflects the intersection of rapid demographic aging, evolving epidemiological profiles, and structural features of China’s health system. Data from the GBD Neurology Collaborators indicate that age-standardized prevalence and disability rates in China have risen at a pace exceeding that of high-income Asia-Pacific countries such as Japan and South Korea (17). These latter countries—despite experiencing advanced population aging—have maintained relatively stable rates, a trend linked to early integration of rehabilitation into chronic disease management, robust primary care systems, and widespread community-based service provision (18, 19). By contrast, China’s demographic transition is more compressed, with a rapid and sustained increase in the proportion of older adults. This demographic momentum is compounded by advances in diagnostic capacity—enabled by expanded neuroimaging, specialized stroke units, and the growth of neurological subspecialties—which have improved case ascertainment but also expanded the population identified with long-term functional impairments (20). Survival gains after acute neurological events, such as ischemic stroke and traumatic brain injury, have further contributed to this expansion, underscoring the imperative for comprehensive post-acute and long-term rehabilitation strategies (21).

The disproportionately high growth in YLDs relative to other indicators signals a persistent imbalance between advances in acute care and the slower expansion of rehabilitation services (3, 22). Comparative analyses from the GBD Neurology dataset show that YLDs growth in China has outstripped that in most high-income and peer upper-middle-income countries (22). While reductions in premature mortality are a major achievement, they increase the number of individuals living with chronic disability (11). In this context, our rehabilitation-focused results provide a complementary lens to prior China-specific burden studies by quantifying the downstream service-relevant consequences of disability accumulation. Yet rehabilitation capacity remains heavily centralized, concentrated in tertiary hospitals in urban areas, with limited integration into primary care and community health systems (23). National and multi-province surveys document stark disparities in rehabilitation bed availability and therapist-to-patient ratios, with western and rural provinces lagging far behind eastern urban centers (9, 24).

These geographic inequalities stem from more than just differences in infrastructure; they are rooted in structural determinants. Policy priorities have historically favored large metropolitan tertiary hospitals, which have received disproportionate investment and advanced technology (23). Economic development levels influence both the fiscal capacity of local governments to fund services and the purchasing power of residents to seek care. Professional workforce distribution is similarly skewed, as rehabilitation specialists tend to remain in urban centers offering greater career advancement, higher remuneration, and better research opportunities (25). This concentration of human resources not only widens urban–rural gaps but also limits access in underserved areas, perpetuating inequities in functional outcomes (9, 24). Strategies to address these imbalances must therefore go beyond infrastructure provision, incorporating targeted policy alignment, equitable financing, and incentives for workforce redistribution.

International models offer valuable lessons but require careful adaptation to China’s context. The decentralized, insurance-supported neurorehabilitation systems of Germany and Japan demonstrate the value of embedding rehabilitation within universal coverage frameworks and linking acute, post-acute, and community-based services (18, 26). However, China’s vast and diverse geography, marked by uneven infrastructure, regional economic heterogeneity, and wide variation in workforce availability, limits the feasibility of direct transplantation (23). For metropolitan and economically advanced regions, earlier transition from acute hospital care to community rehabilitation, supported by long-term care insurance, may be feasible in the near term (18, 26). In less resourced provinces, phased implementation incorporating tele-rehabilitation, mobile outreach, and hybrid public–private partnerships may offer more realistic pathways (27). Adapting financing and governance mechanisms to local administrative capacity will be essential for sustainability (23).

Cause-specific trends reveal important shifts in China’s neurological rehabilitation landscape. Parkinson’s disease has shown some of the steepest increases in both prevalence and incidence in East Asia, with China’s trajectory exceeding that of Japan, South Korea, and Singapore (21, 22). These trends are influenced by both non-modifiable factors (aging) and modifiable factors (pesticide exposure, air pollution, sedentary lifestyles), as well as enhanced diagnostic capacity and clinical awareness (28). Alzheimer’s disease and other dementias have likewise increased at a faster rate in China than in most regional peers, reflecting a combination of longer life expectancy, a rising prevalence of vascular and metabolic risk factors, and more widespread detection via primary care and specialist services (22, 29, 30). Consistent with earlier burden profiles reported in China (6), these neurodegenerative conditions contribute an increasing share of disability; our findings further suggest that their associated rehabilitation needs—particularly for cognitive and functional support—are likely to expand disproportionately in the coming decades. Yet rehabilitation tailored to cognitive disorders—encompassing memory and orientation training, caregiver education, and environmental adaptation—remains scarce, especially in non-urban settings (9, 24).

Trauma-related conditions tell a different story. In many high-income countries, TBI and spinal cord injury incidence has declined substantially due to traffic safety enforcement, occupational hazard mitigation, and robust emergency response systems (23). In China, however, these conditions have declined only modestly, reflecting persistent occupational risks, gaps in injury prevention policies, and incomplete integration of post-acute rehabilitation into trauma care pathways (31). Some early-life neurological conditions, such as cerebral palsy, have increased more slowly in China than globally (22), while neural tube defect prevalence has fallen markedly, coinciding with folic acid supplementation policies and improvements in perinatal care (32).

The age and sex distribution of rehabilitation needs adds further nuance. The burden of degenerative conditions is disproportionately concentrated among older adults, with women showing higher prevalence at advanced ages (29). Biological mechanisms—including hormonal changes post-menopause and sex-specific genetic risk profiles—interact with social determinants such as widowhood, reduced economic resources, and lower access to formal care (29, 33). Multicentre Chinese studies show that older women in rural areas are less likely to receive timely post-acute rehabilitation due to inadequate transport infrastructure, household financial constraints, and limited local service availability (9). In contrast, working-age men exhibit higher rates of trauma-related disabilities, driven by occupational hazards in construction, agriculture, and transportation sectors, as well as risk-related behaviors (23). Evidence supports the effectiveness of gender-responsive interventions—such as tailored fall-prevention and cognitive rehabilitation programs for older women, and strengthened workplace safety enforcement, rapid trauma rehabilitation, and return-to-work schemes for men—in reducing disability and narrowing inequities (33, 34).

The social and economic ramifications of rising neurological rehabilitation needs are considerable. At the household level, caregiving responsibilities frequently fall on family members—disproportionately women—who may withdraw from the workforce, forgo income, and experience psychological strain (24). Out-of-pocket costs for rehabilitation, assistive devices, and home modifications can impose substantial financial burdens, particularly in the absence of comprehensive coverage (24, 35). At the macroeconomic level, insufficient rehabilitation provision can lead to early retirement, loss of skilled labor, and diminished productivity, compounding the challenges of an aging and contracting workforce. Greater demand for rehabilitation will also place strain on public health insurance and social welfare systems, necessitating careful fiscal planning (23). Incorporating cost-effectiveness analyses into rehabilitation policy design could guide resource allocation toward interventions that deliver the greatest functional and economic returns (36).

Projections to 2050 suggest that absolute rehabilitation needs will continue to grow, with Parkinson’s disease, multiple sclerosis, and motor neuron disease showing the steepest increases. This forward-looking evidence complements prior China-focused burden studies by translating observed disability expansion into actionable expectations of future rehabilitation demand, thereby supporting longer-horizon planning for workforce, financing, and service delivery. Without targeted intervention, capacity gaps will widen, waiting times will lengthen, and caregiver burden will intensify. For Parkinson’s disease, under-addressed modifiable risk factors such as pesticide exposure, physical inactivity, and delayed rehabilitation initiation should be prioritized (28). Early-phase multidisciplinary rehabilitation, integrated fall-prevention, and ongoing therapy could help slow progression of disability (33, 37). For multiple sclerosis, key gaps include uneven access to timely diagnosis, limited availability of disease-modifying therapies, and inadequate long-term rehabilitation coverage—especially in rural settings. Tele-rehabilitation platforms and specialist outreach could mitigate these inequities (27, 38). For motor neuron disease, which demands high-intensity multidisciplinary care, strategies should focus on integrating assistive technologies, respiratory support, nutritional management, and caregiver training into community-level services (39).

Incorporating disease-specific rehabilitation pathways into China’s universal health coverage and long-term care insurance frameworks could help close these gaps, but significant implementation challenges remain. Current reimbursement systems are largely oriented toward acute, procedure-based care; they will require revision to accommodate extended, multidisciplinary rehabilitation (23). Fiscal disparities across provinces risk uneven benefit distribution, while administrative fragmentation between health, civil affairs, and social security agencies could impede coordinated care delivery. Policy solutions may include piloting integrated models in selected provinces, linking payment to functional outcome measures, and providing central government subsidies for under-resourced regions. Tele-rehabilitation and mobile health interventions could bridge geographical barriers and help ensure equitable access (27).

In conclusion, China’s rising neurological rehabilitation needs represent a multifaceted challenge spanning clinical, demographic, economic, and systemic domains. Meeting this challenge will require integrated policy reforms to strengthen infrastructure, decentralize services, expand and redistribute the rehabilitation workforce, and embed rehabilitation across the continuum of care. International experience, adapted to local realities, underscores the feasibility of coordinated strategies that combine early detection, comprehensive post-acute intervention, and sustained community support (18, 26). Without such action, the projected trajectory will exacerbate inequities, increase disability, and strain health and social care systems (9). Proactive investment now offers the best opportunity to preserve functional independence, productivity, and quality of life for millions of Chinese adults in the decades ahead (27, 36).

This study has notable strengths, including the use of the Global Burden of Disease 2021 dataset, a specific focus on China’s rapidly aging context, and the application of Bayesian age–period–cohort modeling to generate robust projections. However, limitations must be acknowledged. First, reliance on secondary data modeling may introduce bias, particularly in rural areas with sparse surveillance data. Second, this analysis quantifies epidemiological burden but does not measure rehabilitation service availability, quality, or equity. Third, projections assume static policy and healthcare delivery conditions, without incorporating future innovations or systemic reforms (e.g., tele-rehabilitation, home-based models). Fourth, rehabilitation needs were inferred from disease prevalence and disability, without direct measurement of functional outcomes or quality of life.

## Conclusion

This study demonstrates a substantial rise in the rehabilitation needs for neurological disorders in China from 1990 to 2021, outpacing global averages in both prevalence and disability. The burden is especially pronounced among older adults and females, with notable increases in degenerative conditions such as Parkinson’s disease and Alzheimer’s disease. These trends are expected to intensify by 2050, underscoring the urgent need to scale up rehabilitation services tailored to neurological conditions. Our findings call for integrating rehabilitation into all levels of China’s health system, prioritizing age- and sex-specific strategies, and strengthening policy responses to ensure functional health in an aging society.

## Data Availability

Publicly available datasets were analyzed in this study. This data can be found here: GBD study 2021 data resources were available online from the Global Health Data Exchange (GHDx) query tool (https://vizhub.healthdata.org/gbd-results/).
