# Supplementary material for: Trends in rehabilitation needs for neurological disorders in China, 1990–2021: a cross-sectional analysis of the Global Burden of Disease Study 2021
Source: Front Med (Lausanne). 2026 Apr 9;13:1688298. doi: 10.3389/fmed.2026.1688298 (PMC13102606; doi:10.3389/fmed.2026.1688298)
Supplement: Supplementary file 1 [file Table_1.docx]

**Table legend**

**Table S1. The number of YLDs cases and the age-standardized YLDs rate of rehabilitation needs for neurological disorders in 1990 and 2021, and its temporal trends from 1990 to 2021 in China and Global.** Abbreviations: YLDs, years lived with disability; EAPC, estimate annual percentage change.

**Table S2. The number of prevalence cases and ASPR of rehabilitation needs for neurological disorders by sex in 1990 and 2021, and its temporal trends from 1990 to 2021 in China.** Abbreviations: ASPR = age-standardized prevalence rate, EAPC = estimate annual percentage change.

**Table S3. The number of YLDs cases and age-standardized YLDs rate of rehabilitation needs for neurological disorders by sex in 1990 and 2021, and its temporal trends from 1990 to 2021 in China.** Abbreviations: YLDs = Years Lived with Disability, EAPC = estimate annual percentage change.

Table S1. The number of YLDs cases and the age-standardized YLDs rate of rehabilitation needs for neurological disorders in 1990 and 2021, and its temporal trends from 1990 to 2021 in China and Global. Abbreviations: YLDs, years lived with disability; EAPC, estimate annual percentage change.

| Location |  | 1990 | | 2021 | | 1990-2021 |
| --- | --- | --- | --- | --- | --- | --- |
|  | Disease | Number of cases (95%UI) | Age-standardized YLDs rate /100000(95% UI) | Number of cases (95%UI) | Age-standardized YLDs rate /100000(95% UI) | EAPC (95%CI) |
| **Global** | Neurological disorders | 26648163 (19181210, 34604754) | 608.80 (439.13, 788.52) | 52346199 (37572115, 67457539) | 640.50 (459.39, 824.44) | 0.17 (0.15, 0.19) |
|  | Cerebral palsy | 6365163 (4350789, 8800526) | 112.34 (76.87, 155.22) | 13417691 (9217029, 17953113) | 175.14 (120.28, 234.45) | 1.62 (1.57, 1.68) |
|  | Cerebrovascular disease (stroke) | 7701031 (5516904, 9855454) | 185.31 (132.77, 236.31) | 14675230 (10492831, 18789828) | 172.50 (123.29, 220.39) | -0.32 (-0.34, -0.30) |
|  | Traumatic brain injury | 3590358 (2532780, 4830321) | 77.27 (54.52, 103.67) | 5480354 (3870216, 7331092) | 64.76 (45.75, 86.69) | -0.68 (-0.72, -0.64) |
|  | Alzheimer's disease and dementia | 4836120 (3365347, 6357489) | 150.23 (104.20, 195.07) | 12614239 (8692562, 16499052) | 154.18 (106.42, 201.45) | 0.01 (-0.01, 0.03) |
|  | Spinal cord injury | 3532432 (2494013, 4600912) | 71.78 (50.99, 93.01) | 4566237 (3195694, 6007036) | 54.62 (38.20, 71.97) | -0.73 (-0.77, -0.69) |
|  | Parkinson's disease | 434778 (299127, 587272) | 11.80 (8.18, 15.88) | 1612693 (1108979, 2153494) | 18.94 (13.09, 25.27) | 1.52 (1.49, 1.54) |
|  | Multiple sclerosis | 259747 (181757, 341626) | 5.72 (4.03, 7.53) | 483883 (343395, 629477) | 5.69 (4.03, 7.40) | 0.09 (0.05, 0.12) |
|  | Motor-neuron disease | 34180 (23689, 46867) | 0.71 (0.49, 0.96) | 57476 (40407, 77538) | 0.70 (0.49, 0.95) | 0.11 (0.04, 0.19) |
|  | Guillain-Barré syndrome | 28679 (18067, 43235) | 0.57 (0.37, 0.86) | 139409 (90336, 202013) | 1.75 (1.12, 2.53) | 0.95 (0.26, 1.64) |
|  | Neural tube defects | 285400 (195387, 394596) | 4.84 (3.31, 6.68) | 318997 (220266, 436961) | 4.32 (2.99, 5.91) | -0.37 (-0.41, -0.33) |
| **China** | Neurological disorders | 5112888 (3672585, 6619027) | 572.88 (412.68, 737.27) | 12413299 (8907554, 16153250) | 667.36 (479.14, 869.11) | 0.40 (0.36, 0.43) |
|  | Cerebral palsy | 817055 (553512, 1194835) | 66.36 (44.97, 97.17) | 1144142 (801112, 1530415) | 86.45 (60.48, 115.57) | 1.19 (1.07, 1.32) |
|  | Cerebrovascular disease (stroke) | 2007368 (1420003, 2587433) | 221.53 (155.94, 284.41) | 4937380 (3491196, 6427592) | 245.12 (172.74, 317.42) | 0.33 (0.30, 0.37) |
|  | Traumatic brain injury | 750769 (524411, 1023101) | 70.83 (49.48, 96.57) | 1373267 (962256, 1872386) | 71.53 (49.83, 97.40) | -0.13 (-0.26, -0.00) |
|  | Alzheimer's disease and dementia | 897129 (616784, 1185785) | 158.70 (109.28, 208.07) | 3900585 (2714721, 5165292) | 207.27 (144.04, 271.71) | 0.50 (0.42, 0.59) |
|  | Spinal cord injury | 594359 (417490, 760095) | 52.09 (36.86, 66.42) | 751837 (533035, 983599) | 41.61 (29.28, 54.36) | -1.20 (-1.49, -0.90) |
|  | Parkinson's disease | 91678 (61561, 126021) | 12.66 (8.54, 17.40) | 703410 (483179, 951088) | 33.78 (23.35, 45.84) | 3.14 (3.00, 3.28) |
|  | Multiple sclerosis | 5041 (3251, 7245) | 0.44 (0.29, 0.62) | 11313 (7507, 15962) | 0.63 (0.41, 0.89) | 0.95 (0.79, 1.11) |
|  | Motor-neuron disease | 5427 (3557, 7709) | 0.45 (0.30, 0.64) | 7041 (4730, 9734) | 0.49 (0.32, 0.69) | 0.28 (0.22, 0.35) |
|  | Guillain-Barré syndrome | 1894 (1139, 2960) | 0.17 (0.11, 0.26) | 2779 (1698, 4246) | 0.18 (0.11, 0.29) | 0.39 (0.20, 0.57) |
|  | Neural tube defects | 30307 (19759, 42604) | 2.50 (1.64, 3.52) | 20393 (13858, 28387) | 1.89 (1.30, 2.64) | 0.11 (-0.57, 0.79) |

Table S2. The number of prevalence cases and ASPR of rehabilitation needs for neurological disorders by sex in 1990 and 2021, and its temporal trends from 1990 to 2021 in China. Abbreviations: ASPR = age-standardized prevalence rate, EAPC = estimate annual percentage change.

| Sex | Disease | 1990 | | 2021 | | 1990-2021 |
| --- | --- | --- | --- | --- | --- | --- |
|  |  | Number of cases (95%UI) | ASPR /100000(95% UI) | Number of cases (95%UI) | ASPR /100000(95% UI) | EAPC (95%CI) |
| **Male** | Neurological disorders | 10973714 (10412600, 11637850) | 25463766 (24162641, 26668584) | 2379.69 (2254.24, 2508.04) | 2845.64 (2711.73, 2976.81) | 0.54 (0.52, 0.57) |
|  | Cerebral palsy | 2165293 (1759531, 2748266) | 3119050 (2874838, 3359363) | 338.18 (274.73, 429.32) | 458.93 (423.30, 494.89) | 1.30 (1.20, 1.41) |
|  | Cerebrovascular disease (stroke) | 2889088 (2686725, 3130445) | 7881998 (7231365, 8596483) | 687.82 (632.63, 756.42) | 803.41 (742.92, 872.77) | 0.57 (0.52, 0.62) |
|  | Traumatic brain injury | 3308416 (3166203, 3474354) | 6175428 (5882482, 6512504) | 608.70 (583.12, 638.93) | 641.46 (611.49, 674.04) | 0.04 (-0.07, 0.16) |
|  | Alzheimer's disease and dementia | 1592263 (1350767, 1831599) | 6490156 (5467299, 7436033) | 586.91 (507.03, 674.61) | 753.60 (642.39, 863.24) | 0.55 (0.48, 0.61) |
|  | Spinal cord injury | 1058827 (982157, 1152655) | 1735104 (1605012, 1877031) | 180.02 (167.14, 195.17) | 188.11 (173.81, 204.82) | -0.15 (-0.38, 0.07) |
|  | Parkinson's disease | 153373 (125827, 185674) | 1350027 (1105370, 1641355) | 49.00 (39.78, 59.26) | 141.44 (116.71, 172.08) | 3.44 (3.31, 3.58) |
|  | Multiple sclerosis | 6091 (4463, 8119) | 13252 (10372, 16471) | 1.05 (0.78, 1.36) | 1.45 (1.12, 1.84) | 0.93 (0.80, 1.05) |
|  | Motor-neuron disease | 11405 (9041, 14247) | 14581 (11826, 17613) | 1.83 (1.48, 2.25) | 2.01 (1.60, 2.43) | 0.35 (0.29, 0.41) |
|  | Guillain-Barré syndrome | 3464 (2517, 4707) | 4903 (3615, 6442) | 0.62 (0.46, 0.81) | 0.64 (0.48, 0.84) | 0.28 (0.10, 0.46) |
|  | Neural tube defects | 46405 (35663, 58791) | 31576 (26298, 37631) | 7.34 (5.64, 9.31) | 5.53 (4.59, 6.62) | 0.06 (-0.54, 0.67) |
| Female | Neurological disorders | 9872262 (9331012, 10413473) | 25086220 (23583263, 26630488) | 2220.33 (2095.67, 2346.93) | 2549.12 (2410.59, 2695.38) | 0.30 (0.26, 0.34) |
|  | Cerebral palsy | 1652309 (1374168, 2040216) | 2264608 (2102586, 2438074) | 279.73 (232.55, 345.72) | 345.84 (321.04, 372.53) | 1.08 (0.93, 1.23) |
|  | Cerebrovascular disease (stroke) | 3440607 (3196396, 3718733) | 8441991 (7629189, 9237886) | 745.26 (687.51, 807.84) | 809.52 (736.53, 880.57) | 0.20 (0.13, 0.26) |
|  | Traumatic brain injury | 1665216 (1600089, 1742095) | 3094152 (2940444, 3260023) | 329.18 (315.73, 344.44) | 316.75 (301.85, 332.87) | -0.34 (-0.49, -0.18) |
|  | Alzheimer's disease and dementia | 2634417 (2276624, 2978640) | 11222191 (9794176, 12653418) | 802.04 (701.60, 907.26) | 1053.44 (922.37, 1189.29) | 0.50 (0.42, 0.59) |
|  | Spinal cord injury | 634569 (586322, 689491) | 1029261 (933774, 1134766) | 116.90 (108.30, 126.38) | 113.24 (102.87, 125.65) | -0.61 (-0.93, -0.28) |
|  | Parkinson's disease | 147745 (120466, 178852) | 992652 (827131, 1201246) | 38.34 (31.32, 45.94) | 90.32 (75.66, 109.57) | 2.75 (2.64, 2.86) |
|  | Multiple sclerosis | 7675 (5744, 10151) | 17652 (14137, 21909) | 1.36 (1.04, 1.76) | 1.99 (1.57, 2.55) | 0.95 (0.76, 1.15) |
|  | Motor-neuron disease | 10657 (8449, 13259) | 14051 (11274, 17156) | 1.83 (1.48, 2.25) | 1.93 (1.54, 2.36) | 0.21 (0.13, 0.29) |
|  | Guillain-Barré syndrome | 2935 (2158, 3988) | 4483 (3321, 5862) | 0.54 (0.41, 0.71) | 0.59 (0.45, 0.78) | 0.49 (0.30, 0.69) |
|  | Neural tube defects | 41203 (31314, 53765) | 27250 (22355, 32917) | 7.12 (5.44, 9.27) | 5.38 (4.42, 6.46) | 0.16 (-0.62, 0.94) |

Table S3. The number of YLDs cases and age-standardized YLDs rate of rehabilitation needs for neurological disorders by sex in 1990 and 2021, and its temporal trends from 1990 to 2021 in China. Abbreviations: YLDs = Years Lived with Disability, EAPC = estimate annual percentage change.

| Sex | Disease | 1990 | | 2021 | | 1990-2021 |
| --- | --- | --- | --- | --- | --- | --- |
|  |  | Number of cases (95%UI) | Age-standardized YLDs rate /100000(95% UI) | Number of cases (95%UI) | Age-standardized YLDs rate /100000(95% UI) | EAPC (95%CI) |
| **Male** | Neurological disorders | 2631309 (1889090, 3404528) | 6124766 (4380613, 7962877) | 577.02 (416.60, 746.16) | 684.71 (491.53, 890.73) | 0.52 (0.50, 0.55) |
|  | Cerebral palsy | 463450 (313436, 688072) | 663010 (463651, 889958) | 72.35 (48.93, 107.31) | 97.83 (68.39, 131.29) | 1.29 (1.18, 1.40) |
|  | Cerebrovascular disease (stroke) | 930555 (653536, 1201557) | 2440468 (1743065, 3181533) | 217.72 (154.02, 281.04) | 248.75 (177.51, 324.42) | 0.50 (0.45, 0.56) |
|  | Traumatic brain injury | 503113 (349600, 687676) | 922488 (646311, 1262337) | 91.77 (63.87, 125.23) | 96.09 (67.02, 131.25) | 0.02 (-0.09, 0.14) |
|  | Alzheimer's disease and dementia | 316057 (218011, 411872) | 1327305 (926845, 1761937) | 121.75 (83.07, 161.79) | 158.43 (110.15, 211.36) | 0.58 (0.51, 0.65) |
|  | Spinal cord injury | 377883 (266194, 488067) | 482612 (342310, 634900) | 63.69 (45.11, 81.93) | 52.70 (37.23, 69.48) | -1.00 (-1.26, -0.75) |
|  | Parkinson's disease | 47270 (31371, 64876) | 409953 (276864, 557146) | 14.70 (10.00, 20.09) | 42.45 (29.11, 57.38) | 3.42 (3.28, 3.56) |
|  | Multiple sclerosis | 2231 (1406, 3232) | 4851 (3196, 6871) | 0.38 (0.24, 0.55) | 0.53 (0.35, 0.75) | 0.92 (0.80, 1.05) |
|  | Motor-neuron disease | 2806 (1858, 4018) | 3586 (2399, 4924) | 0.45 (0.30, 0.63) | 0.50 (0.33, 0.70) | 0.35 (0.29, 0.40) |
|  | Guillain-Barré syndrome | 1025 (606, 1603) | 1452 (886, 2233) | 0.18 (0.11, 0.28) | 0.19 (0.12, 0.30) | 0.28 (0.10, 0.46) |
|  | Neural tube defects | 16045 (10364, 22631) | 10946 (7395, 15338) | 2.54 (1.65, 3.57) | 1.92 (1.31, 2.68) | 0.07 (-0.53, 0.67) |
| Female | Neurological disorders | 2481578 (1782658, 3186391) | 6288533 (4538268, 8203175) | 559.49 (402.97, 720.00) | 638.11 (460.01, 830.80) | 0.27 (0.22, 0.32) |
|  | Cerebral palsy | 353605 (240973, 506808) | 481132 (336751, 639351) | 59.84 (40.78, 85.84) | 73.72 (51.38, 98.08) | 1.06 (0.91, 1.20) |
|  | Cerebrovascular disease (stroke) | 1076812 (766906, 1387615) | 2496912 (1759772, 3241350) | 229.92 (163.34, 294.84) | 241.73 (169.41, 312.94) | 0.09 (0.04, 0.15) |
|  | Traumatic brain injury | 247656 (174364, 334443) | 450779 (317384, 611438) | 48.58 (34.19, 65.69) | 46.39 (32.46, 63.37) | -0.36 (-0.51, -0.21) |
|  | Alzheimer's disease and dementia | 581072 (401775, 767171) | 2573279 (1784413, 3382028) | 182.57 (126.39, 241.65) | 243.49 (168.37, 320.45) | 0.53 (0.44, 0.63) |
|  | Spinal cord injury | 216476 (152430, 272711) | 269225 (187753, 354335) | 39.54 (28.11, 49.94) | 29.97 (20.90, 39.75) | -1.50 (-1.85, -1.14) |
|  | Parkinson's disease | 44408 (29858, 61474) | 293457 (202034, 398189) | 11.37 (7.68, 15.63) | 26.60 (18.40, 36.04) | 2.72 (2.60, 2.84) |
|  | Multiple sclerosis | 2811 (1843, 4053) | 6462 (4257, 9054) | 0.50 (0.33, 0.71) | 0.73 (0.48, 1.04) | 0.95 (0.75, 1.14) |
|  | Motor-neuron disease | 2622 (1699, 3723) | 3456 (2327, 4786) | 0.45 (0.30, 0.63) | 0.47 (0.31, 0.68) | 0.21 (0.13, 0.29) |
|  | Guillain-Barré syndrome | 869 (528, 1366) | 1327 (819, 2016) | 0.16 (0.10, 0.25) | 0.18 (0.11, 0.27) | 0.49 (0.30, 0.69) |
|  | Neural tube defects | 14262 (9368, 20324) | 9447 (6388, 13151) | 2.47 (1.62, 3.50) | 1.86 (1.27, 2.61) | 0.16 (-0.61, 0.94) |
